# Estimates of the Economic Cost Caused by Five Major Reproductive Problems in Dairy Animals in Assam and Bihar, India

**DOI:** 10.3390/ani11113116

**Published:** 2021-10-30

**Authors:** Ram Pratim Deka, Ulf Magnusson, Delia Grace, Thomas F. Randolph, Rajeswari Shome, Johanna F. Lindahl

**Affiliations:** 1 Department of Clinical Sciences, Swedish University of Agricultural Sciences (SLU), 75007 Uppsala, Sweden; ulf.magnusson@slu.se (U.M.); J.Lindahl@cgiar.org (J.F.L.); 2 International Livestock Research Institute (ILRI), Nairobi 00100, Kenya; d.grace@cgiar.org (D.G.); t.randolph@cgiar.org (T.F.R.); 3 Natural Resources Institute, University of Greenwich, Kent ME4 4TB, UK; 4 Indian Council of Agricultural Research, National Institute of Veterinary Epidemiology & Disease Informatics (ICAR-NIVEDI), Bangalore 560064, India; rajeswarishome@gmail.com; 5 Department of Medical Biochemistry and Microbiology, Uppsala University, 75123 Uppsala, Sweden

**Keywords:** reproductive problems, dairy animals, economic burden, cost, loss, India

## Abstract

**Simple Summary:**

Large ruminant dairy animals (i.e., cattle and buffalo) suffer from several reproductive problems (such as abortion) that reduce ther ability to produce milk and offspring, resulting in huge economic costs to farmers; however, there are few studies in India that estimate such costs. Therefore, an attempt was made to assess the economic cost of five major reproductive problems in two of the poorest Indian states—Assam and Bihar. We estimated the cost by interviewing 534 randomly selected dairy farming households in both the states. Based on this, we found that 32.9% of dairy animals (milking, not-milking and heifer) in Assam and 43.1% dairy animals in Bihar suffered from one or more reproductive problems. The most common reproductive problem was failing to conceive after breeding (23.2% of surveyed dairy animals) followed by retained placenta (6.1%), abortion (4.9%), purulent vaginal discharge (2.9%) and stillbirths (1.0%). It was estimated that the selected reproductive problems caused an annual economic cost of Indian Rupees (INR), 3963.1 million (USD 59.0 million) in Assam, and INR 30,500.0 million (USD 453.9 million) in Bihar. The study concludes that adequate awareness, capacity building, adoption of good reproductive health management practices, proper farm record keeping and improved access to quality veterinary services are essential to address reproductive problems and reduce the cost caused by these reproductive problems.

**Abstract:**

Reproductive problems in dairy animals reduce fertility, prevent conception, create problems in the delivery of healthy calves, lead to postpartum complications, increase inter-calving periods, reduce milk yield, and lower overall lifetime productivity. This study aimed at understanding the incidence of reproductive problems and the cost caused by these. The study covered 954 dairy animals in Bihar and 1348 dairy animals in Assam that were selected using a multi-stage random sampling method. The costs were calculated as the sum of income losses and expenditures incurred. The major cost incurred resulted from extended calving intervals (46.1% of the total cost), followed by loss through salvage selling (38.1%), expenditure for treatment of repeat breeders (5.9%), loss of milk production (5.3%) and expenditure for extra inseminations (2.0%). About one fifth of the selected reproductive problems were left untreated. The estimated cost of reproductive problems was Indian Rupees (INR) 2424.9 (USD 36.1) per dairy animal per year (of the total dairy animal population) which represented approximately 4.1% of the mean value loss of dairy animals (INR 58,966/USD 877) per year. Reproductive problems were significantly (*p* < 0.001) higher among improved (exotic breed or cross-bred) dairy animals than indigenous (native breed or nondescript indigenous) dairy animals. The study suggests that with the increase of improved dairy animal population, the loss may further increase. The study concludes that any economic estimation of reproduction problems based on aetiology without confirmatory diagnoses could be highly misleading because of the complex nature of the problems.

## 1. Introduction

India is the world’s largest producer of milk, producing 194.8 million tons in 2020 out of a global production of 906.0 million tonnes [1]. India’s annual milk production grew by 6.5% in 2018–19 (April–March) compared to the annual growth of 1.4% global growth [2,3]. In the same year, the livestock sector in India contributed about 4.9% of the total Gross Domestic Product (GDP) of the country, or 28% of the total output value of the agricultural sector [2]. India also has the largest bovine population in the world with 193.5 million cattle and 109.8 million buffaloes as per the livestock census held in 2019 [2]. High milk output is more because of high numbers of dairy animals rather than high productivity: the opposite of the situation in many other developed countries [4,5]. One of the main reasons for lower productivity in India is a high prevalence of reproductive problems [6,7] which have an important bearing on productive and reproductive performance as well as farm economics. Among the reproductive problems commonly reported are: repeat breeding, anoestrus, retained placenta, dystocia, abortion, stillbirth, purulent vaginal discharge and uterine prolapse [4,8,9]. These reproductive problems reduce fertility, prevent conception, create problems in the delivery of healthy calves, lead to postpartum complications, increase inter-calving periods, reduce milk yield and lower overall lifetime productivity [9,10]. Further, to manage these problems, farmers need to spend money for treatment (buying medicine and paying for animal health provider fees) and to manage (feed, house, labour, water, electricity, etc.) dairy animals during a longer unproductive inter-calving period, contributing to higher cost and reducing profits. On many occasions, treatments fail, and farmers may be compelled to sell the animals at a reduced price before the end of their productive life (salvage selling) generating further economic loss. Cow slaughter is subject to legal prohibitions and restrictions in several states in India for socio-religious reasons. Slaughtering of cows for meat purposes is restricted in Assam under the “Assam Cattle Preservation Act, 1950” and in Bihar under the “Bihar Preservation and Improvement of Animal Act, 1955”. Therefore, unproductive cows do not carry much value in either state. Large numbers of unproductive beef cattle are informally traded (smuggled) to Bangladesh where demand for beef is very high and they can fetch a higher price than in India [11].

Estimating the cost of reproductive problems requires assessing their physical effects on dairy animals and expressing these in terms of economic cost. It is difficult to quantify the exact costs as their effects are not always certain and these may be influenced by other factors (e.g., breed, feed, healthcare, management, stage of occurrence, severity of disease, etc.) or may manifest with other diseases [12]. In addition, effects may last from days to years which adds to the difficulty of cost estimation. Absence of data recording systems, especially in smallholder dairy farms, further increases the challenge of characterizing reproductive health [13].

As a result of the complexity of the subject, lack of accessible farm data and requirement of laboratory investigation to diagnose some of the reproductive problems, there is a paucity of economic studies on the reproductive health of dairy animals in India. This type of study is important to understand the extent of the problem and to inform policy makers about the need for investment to control the problem. Therefore, an attempt was made to assess the economic cost caused by reproductive problems; however, this paper does not analyze the economic cost caused by the full range of reproductive problems but focuses on five selected priority reproductive problems namely: abortion, repeat breeding, retained placenta, purulent vaginal discharge, and stillbirth. These problems were selected as they are common in dairy animals in the region [7], and they can easily be identified by the farmers using a syndromic approach without depending on the costly biological sampling and testing procedure, which were beyond the resources available for the study.

## 2. Materials and Methods

### 2.1. Data and Sampling Procedure

This analysis used data collected during a cross-sectional study conducted in two of the poorest Indian states, Assam and Bihar, during 2015–16. The detailed sampling methods for the study are explained elsewhere [14,15]. In short, a survey of 534 dairy farming households was conducted using multi-stage sampling where, at the first stage, three districts were purposefully selected in each state based on their importance in terms of dairy development (classified as high, medium, and low). In the second stage, two community development blocks (CDBs) were randomly selected from each selected district. At the third stage, four villages were randomly selected from the list of villages in each CDB, and then ten households were selected randomly from the list of households having large ruminant dairy animals (cattle or buffalo that are milking, non-milking and heifer) in each selected village. In creating the list of farming households, local informants, local veterinary officers, and village head men helped the study team. They further helped in informing the farming households about the visit of the study team well in advance. The household representatives responsible for management of the dairy herds were interviewed after obtaining their written consent to participate in the study.

The ethical approval for the study was received from the Institutional Research Ethics Committee (IREC) of the International Livestock Research Institute (ILRI) on 21 September 2015 vide letter No. ILRI-IREC2015-12.

A structured pre-tested questionnaire was used for interviewing each farming household with questions related to the background of farmers; farming system; bovine mating system; cost of mating; productive and reproductive performances; occurrence of reproductive problems; loss of milk yield; price of milk; price of feed and fodder; cost of management of dairy animals; extended calving interval, and loss and expenditure incurred by the farmers for management of the animals. Information was also captured about the number of dairy animals suffering from each reproductive problem in each household, number of animals treated, and the type of treatment given by the veterinarians. As most of the farmers could not report the name of the medicines, leftover medicines, if any, were checked and further confirmation were taken from the local veterinary officer who mostly accompanied the study team. Data on reproductive problems were collected based on the experiences of farmers encountering the reproductive problems in the 12 months previous to the date of the survey. The cost of treatment of abortion, the problem of highest interest, was collected in the farm household survey, but costs associated with the four other reproductive problems (i.e., repeat breeding, retained placenta, purulent vaginal discharge, and stillbirth) were collected using a participatory method with a focus group discussion (FGD) that was organized in each selected CDB. In each FGD about 15–20 farmers (in total 121 farmers in 7 FGDs) who participated in our farm survey, and who reported the targeted reproductive problems, took part. The agreed costs of reproductive problems based on their experience of treating the problems during the previous 12 months from the date of FGD were noted for analysis.

As smallholder farmers in Assam and Bihar hardly maintained any farm records, they responded to our questions based on their memories. In order to validate the information/data stated by them, they were asked supplementary questions for inconsistent reports at the time of interview. Further, thorough data cleaning was conducted to remove obvious erroneous responses. If any inconsistency was observed, the respondent was telephoned to correct information. There were some absent data or non-applicable data more particularly related to the cost of management of dairy animals. At the time of data entry, the cell for absent data/non applicable data was left blank (i.e., not recorded as zero) in order to avoid counting of the cell in the statistical analysis. As such, no questionnaire was fully rejected based on one or more absent/non applicable datum. The total number of farming households who responded to relevant questions and who did not respond/were found not applicable to respond were calculated.

The collected data were entered in Microsoft EXCEL and were analyzed using Stata-14 version (STATA Corp Ltd., Texas, TX, USA). The categorical variables were analyzed by using chi-square test while the continuous variables were analyzed using *t*-test. A p-value of 0.05 or less was considered statistically significant. For categorical variables, the percentage of responses out of the corresponding total were calculated while in case of continuous variables, mean value was calculated.

Finally, the costs of managing reproductive problems were estimated using the economic model as indicated in the following section.

### 2.2. Estimating Cost of Reproductive Problems

The total economic cost of reproductive problems was estimated in terms of two distinct components as set out by McInerney et al. (1992): loss and expenditure [16]. A loss (L) implies a benefit that is taken away or alternatively, a potential benefit that is not realized (such as when disease causes milk yield to fall). On the other hand, expenditure (E) represents resources that have to be allocated to unplanned or non-preferred uses (such as treatment of diseased animals, feeding for extra days, etc.). The term economic cost (C) is used to represent the sum of both loss and expenditure. The economic model was developed based on previous studies [17,18,19] with various adaptations as these studies estimated only the cost of brucellosis whereas we estimated the cost of five reproductive problems with unknown etiology.

The following definitions were used to estimate the total economic cost caused by reproductive problems in the surveyed households. This cost estimation was based on all the dairy animals that suffered from one or more reproductive problems in the surveyed households.

### 2.3. Explanation of Key Terminologies Used in This Paper

Different key terminologies of reproductive problems that are used in the study are explained below to avoid any ambiguity in understanding.

Repeat breeding (RB): If a cow cycled normally with no clinical abnormalities but failed to conceive after at least two successive inseminations, this was considered repeat breeding.

Abortion (A): A fetus lost between the age of 42 days and approximately 260 days of pregnancy was considered as an abortion. Pregnancies lost before 42 days were considered as early embryonic deaths [20].

Stillbirth (SB): If a calf was born dead between 260 days and full term (280 days), this was considered as stillbirth.

Purulent vaginal discharge (PVD): Presence of purulent or mucopurulent exudates (cloudy, whitish, yellow, greenish, or bloody discharge) through the female genital tract as a result of infection was considered as purulent vaginal discharge. This broader terminology was used to cover reproductive problems such as metritis and endometritis given the absence of any laboratory investigation to confirm these.

Retained placenta (RP): If a cow failed to expel fetal membranes within 24 h after calving, this was considered as retained placenta.

Calving interval (CI): The time between two successive calvings was considered as the calving interval.

Extended calving interval: Extension of the time period between two successive calvings among the animals who suffered from reproductive problems, mainly because of abortion and repeat breeding, beyond the normal period of successive calvings (15.2 ± 0.1 months) in the given dairy animal population was considered an extended calving interval.

Expenditure for treatment (ET): This included the expenditure on medicine and veterinarian fees that were incurred by farmers for treating dairy animals suffering from reproductive problems.

Large ruminant dairy animals: These included both cattle and buffalo of sexually mature age (milking, non-milking and heifer).

Loss of milk production (LMP): If a milking cow produced less milk for some days or months after abortion than its anticipated milk production, this was considered as loss of milk production.

Livestock salvage sale (LSS): A dairy animal with reproductive problem/s sold at lower price than its normal market value was regarded as salvage sale.

Reproductive month loss: After occurrence of a reproductive problem such as abortion or repeat breeding, a dairy animal lost days or months during which the animal did not gain anything in terms of reproductive cycle, but the farmer continued to spend money on feed, labor, electricity, etc., for management of the animals. The loss incurred during this period was considered as reproductive month loss.

### 2.4. Loss and Expenditure Incurred for Managing Reproductive Problems

The economic model used for estimating the loss and expenditure incurred for reproductive problems is stated below:

Total economic cost caused by reproductive diseases (TEC) = Losses caused by reproductive problems + Expenditure caused by reproductive problems.

TEC = (LMP + LSS) + (ETA + ETRB + ETRP + ETPVD + ETSB + EEI + ERMLA + ERMLRB).

where,

Loss of milk production (LMP): (Number of animals with reduced milk yield after abortion) × MLA (Mean milk loss per animal in litre) × (Number of days with reduced milk yield) × (Mean price of milk per litre).

Loss caused by salvage sale (LSS): (Number of animals sold in salvage) × {(Mean price of animal without disease) − (Mean price of animals with disease)}.

Expenditure for treatment of abortion (ETA)/repeat breeding (ETRB)/retained placenta (ETRP)/purulent vaginal discharge (ETPVD)/stillbirth (ETSB) = (Number of animals treated) × (Mean expenditure of treatment).

Three other expenditures that are incurred by farmers for reproductive problems include:

Expenditure for extra insemination of repeat breeders (EEI) = (Number of repeat breeder animal) × (Mean number of extra inseminations required per repeat breeder) × (Mean expenditure for artificial insemination).

Expenditure for animal management during reproductive month loss for abortion (ERMLA) = (Number of animals that lost reproductive months because of abortion) × (Mean number of months lost for abortion) × (Mean expenditure of rearing per dairy animal per month).

Expenditure for animal management during reproductive month loss for repeat breeding (ERMLRB) = (Number of animals that lost reproductive months because of repeat breeding) × (Mean month loss for repeat breeding) × (Mean expenditure of rearing per dairy animal per month).

To calculate the mean expenditure of rearing per dairy animal per month, the following formula was used: 

Mean expenditure of rearing per dairy animal per month = {(Quantity of concentrate feed consumed/animal/day × price/kg) + (Quantity of fodder consumed/animal/day × price/kg) + (Average expenditure of labor/animal/day) + (Other miscellaneous expenses/animal/day) + (Average expenditure of electricity/animal/day)} × 30 days.

The initial estimates were made following the calculation method above in order to represent the total aggregate cost incurred by the surveyed households in the study locations of Assam and Bihar. We extrapolated the cost from the sample to the whole state by assuming the same unit cost and same percentage of dairy animals that suffered from reproductive problems in our study sites in Assam and Bihar for the respective state. To extrapolate the cost, secondary data of the numbers of mature female dairy animals (milking, non-milking and heifer) available in Assam and Bihar were taken from the National Livestock Census held in 2019 [21]. Dairy animal population data included the total dairy animals irrespective of farm category or rearing system and therefore our estimate did not address the bias, if any, associated with these factors.

The cost of reproductive problems of indigenous (native breed or non-descript indigenous) and improved (exotic or cross bred) dairy animals was estimated separately to account for the different shares of indigenous and improved dairy animal population. In India, purebred dairy animals that are imported from abroad, or descended from these (e.g., Jersey, Holstein Friesian, etc.) are considered as exotic breed, while indigenous animals that are characterized and notified as a breed by the National Bureau of Animal Genetics Resources (NBAGR), Govt. of India, are called indigenous breed, and those native animals that are not characterized and notified as a breed are called non-descript indigenous. 

We considered that the dairy farming prevalent in the three selected districts of each state were largely representative of the whole state and therefore we did not weight the districts. In addition, although prices of dairy animals varied based on breed, age, milk yield, etc., we considered only the mean prices. Similarly, the mean expenditure of treating reproductive problems of indigenous and improved dairy animals was applied to both groups of dairy animals. All estimated costs related to the 12 months period preceding the date of the survey. 

## 3. Results

### 3.1. Description of Sample Households

In total, 292 households in Bihar and 242 households in Assam were surveyed. The surveyed households kept in total 2302 large ruminants (cattle and buffalo) dairy animals of which 1348 were from Assam and the remaining 954 were from Bihar.

Demographic features of the sampled households and dairy animals are described elsewhere [14,15]. In brief, it was found that mean herd size of dairy farms in Assam (4.1) was significantly (*p* < 0.01) higher than in Bihar (2.8), and higher in urban areas (5.5) than in rural areas (2.9). In addition, mean herd sizes were higher in Kamrup (metropolitan) district of Assam and Patna district of Bihar, where the respective major cities (Guwahati and Patna) of Assam and Bihar are located, than in the remaining districts. In Assam, a significantly (*p* < 0.001) higher percentage of farming households (72.3%) reared dairy animals under partly stall-fed conditions than in Bihar (14.8%). Households with fully stall-fed (zero grazing) rearing were concentrated in Kamrup (metropolitan) district (69.1%) in Assam, while in Bihar, distributions of such farming households were almost uniform across the districts. In regard to the breed of dairy animals, 79.1% of the surveyed dairy animals (*n* = 1066) in Assam and 94.4% (*n* = 901) in Bihar were improved (exotic or cross bred) and the remaining were indigenous.

### 3.2. Reproductive Problems at Household Levels

With regard to the reproductive problems at households level, it was found that the five problems of interest (repeat breeding, abortion, retention of placenta, purulent vaginal discharge and stillbirth) were significantly higher (Table 1) in the dairy farms located in urban areas than in rural areas; in large (>10 dairy animals) and medium (4–10 dairy animals) sized farms compared to small-sized (1–3 dairy animals) farms; under a fully stall-fed (zero grazing) system compared to partly stall-fed system; and, in the farms that reared improved animals compared to farms with indigenous animals.

In Assam, the percentage of dairy farming households who encountered one or more of the five reproductive problems in the 12 months prior to the survey was significantly (*p* < 0.001) higher in Kamrup (metropolitan) district (65.4%) than Baksa (11.3%) and Golaghat (7.4%) districts while, in case of Bihar, the percentages of households who encountered reproductive problems during the same period were almost similar in Patna (58.3%), Nalanda (46.5%) and Vaishali (48.2%) districts.

### 3.3. Basic Data Used for Estimation of the Cost of Reproductive Problems

The summary of five selected reproductive problems of dairy animals of surveyed farming households and the parameters essential to estimate the cost of the problems are stated in Table 2. All the cost estimations in the subsequent tables were developed in light of the figures in Table 2. The table indicated the absent data or non-applicable data as well. We found that 32.9% of dairy animals belonging to 28.1% of farming households in Assam and 43.1% of dairy animals belonging to 51.9% of dairy farming households in Bihar suffered from one or more of the selected reproductive problems. Of the affected households, 92.6% dairy farming households in Assam treated reproductive problems while in Bihar only 72.8% farming households treated reproductive problems. In terms of the percentage of animals treated, about one fifth of affected animals were left untreated.

The most common of the five reproductive problems reported in the study area was repeat breeding (23.2%) followed by retained placenta (6.1%), abortion (4.9%), purulent vaginal discharge (2.9%) and stillbirths (1.0%). Among the reproductive problems, percentages of dairy animals experiencing repeat breeding, abortion and stillbirth were significantly higher in Bihar than in Assam, while percentage of dairy animals experiencing retained placenta and purulent vaginal discharge were significantly higher in Assam than in Bihar. In Assam, a larger share of dairy animals aborted in the third trimester than in Bihar.

### 3.4. Estimation of the Cost of Reproductive Problems in the Surveyed Areas

In light of the data presented in Table 2, the economic costs of reproductive problems in the surveyed areas were estimated and presented in Table 3. As incidence of reproductive problems significantly varied between indigenous and improved dairy animals, the cost of all reproductive problems was calculated according to herd composition (indigenous or improved dairy animals).

It was observed that reproductive months lost (due to repeat breeding and abortion) was the single most important problem, comprising nearly half of the total cost of all reproductive problems; this was due to the costs of management of dairy animals (feed, fodder, labour, electricity, etc.). The major cause of reproductive month loss was abortion followed by repeat breeding; both were significantly (*p* < 0.05) higher per dairy farming household in Assam than in Bihar. However, the total number of animals that suffered from abortion and repeat breeding was higher in Bihar than in Assam. It was found that the cost of managing dairy animals per day was relatively higher in Assam than in Bihar. Next to reproductive months lost, the second most important cost was salvage selling loss. This was again higher in Assam than in Bihar as both the number of animals sold for salvage and price of dairy animals were higher (Table 2). Most of the dairy animals in Assam sold for salvage came from Kamrup (Metropolitan) district, where most dairy farmers reared larger herds under fully stall-fed conditions than in the other two districts. Similarly, the average loss of milk per animal, and the number of animals with milk loss was also higher in Assam than in Bihar. The average price of milk per litre was about 35% higher in Assam than in Bihar. The same was the case for extra insemination requirements (more than two inseminations) per repeat breeder and per unit expenditure of insemination. All the treatment expenditures were significantly higher in Assam than in Bihar.

### 3.5. Extrapolation of the Cost of Reproductive Problems for the State of Assam and Bihar

In order to extrapolate the cost of reproductive problems to the whole state of Assam and Bihar, we estimated the affected dairy animal population for both Assam and Bihar (Table 4) following the approach explained in the methodology section. The affected dairy animals in each state were estimated based on the total number of dairy animals (in milk, dry and heifer) in Assam (*n* = 3,738,775) and Bihar (*n* = 10,473,230) as per the 20th livestock census conducted in 2019. In Assam, 92.2% (*n* = 3,445,483) of dairy animals were indigenous and the remaining 7.8% (*n* = 293,292) were improved, while in Bihar, 80.4% (*n* = 8,419,959) were indigenous and the remaining 19.6% (*n* = 2,053,271) were improved. In Bihar, the total dairy animal population was 73.7% higher than in Assam, and the improved dairy animal population was 87.5% higher than in Assam.

**Figure 1:**
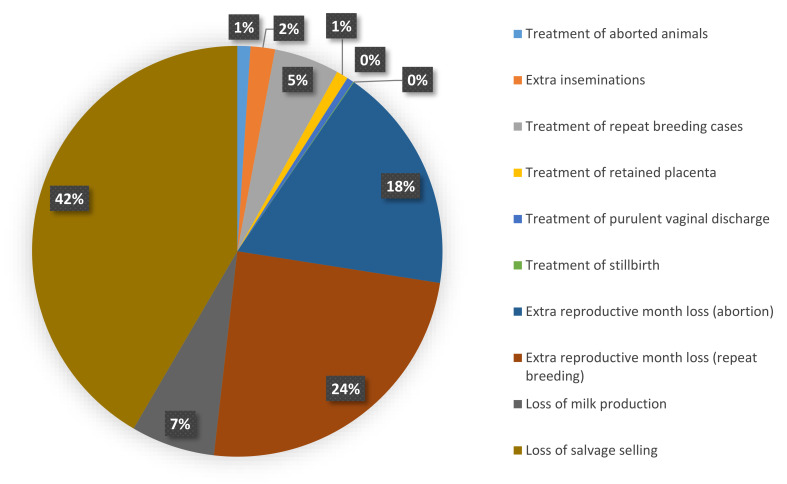
Distribution of different cost components to the total reproductive cost in Assam.

**Figure 2:**
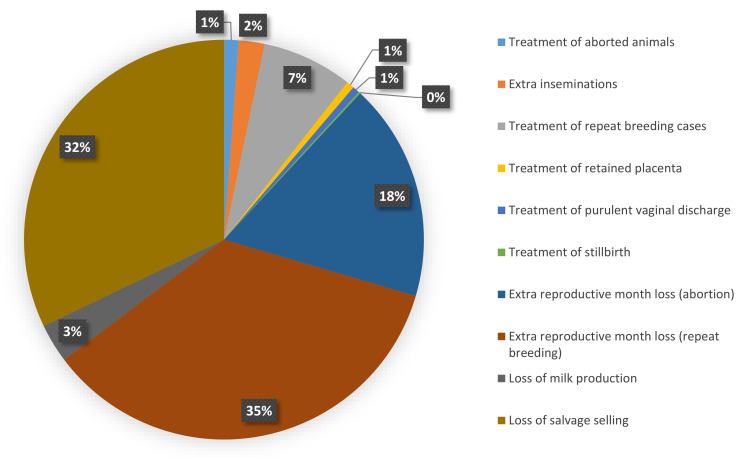
Distribution of different cost components to the total reproductive cost in Bihar.

Because of the higher unit cost of each reproductive problem per animal in Assam compared to Bihar (Table 2), the cost per affected animal was higher in Assam (Table 3). Despite this, the extrapolated cost of reproductive problems per dairy animal per year (Table 5) was about three times higher in Bihar (INR 2912/USD 43.3) than in Assam (INR 1060.0/USD 15.8). The average cost per dairy animal per year including both the states was INR 2424.9 (USD 36.1). The percentage of cost contributed by different cost components to the total estimated cost for the state of Assam and Bihar are presented in Figure 1 and Figure 2.

The total cost of reproductive problems in Assam was just 13.0% of the total cost in Bihar. Little more than half (52.4%) of the cost of reproductive problems in Assam was due to indigenous dairy animals, while in Bihar about 61.9% of the cost was due to indigenous dairy animals.

The estimated cost of INR 2424.9 (USD 36.1) per dairy animal of the total dairy animal population represents approximately 4.1% of the mean value of dairy animals (INR 58,966/USD 877).

## 4. Discussion

### 4.1. Prevalence of Reproductive Problems and Factors Associated with These

As noted in the preceding section, the prevalence of the five priority reproductive problems varied according to a number of factors. It was found that the selected reproductive problems were widely present in dairy animals of Assam and Bihar, with prevalence varying within and between states. The prevalence of dairy animals with one of more of the selected reproductive problems found in our study in Assam (32.9%) and in Bihar (43.1%) align well with an earlier study in Meghalaya, neighbouring Assam, which reported that 33.8% of dairy animals in the state were affected by one or more reproductive problems [7]. A study in Bangladesh, which borders Assam, also reported a similar prevalence (39.4%) of reproductive problems [22], as did studies conducted in Kashmir, India (41.8%) [23] and in Ethiopia (43.1%) [4]. On the other hand, a study in Afghanistan reported a much higher prevalence (55.6%) [24].

There is much debate in the development literature over the relative challenges and opportunities offered by smallholder, traditional, less intensive systems versus larger, modern, more intensive systems. We found that prevalence of the selected reproductive problems in improved dairy animals was significantly (*p* < 0.001) higher than in indigenous animals. A similar finding was reported in a study in Ethiopia [25]. Indigenous animals might be expected to suffer fewer reproductive problems given their lower reproductive efficiency relative to improved dairy animals [25,26], lower use of artificial insemination reducing the chances of repeat breeding because of issues such as poor semen quality, improper timing or faulty insemination, as well as being better adapted to local climatic conditions that might make them more tolerant of or resistant to various reproductive diseases. Further, this may also be attributed to farmers not recognizing or paying attention to the reproductive problems in indigenous animals because of their generally poor productive and reproductive performance and because they are often kept in open grazing systems, especially in Assam.

The average reproductive problems per farming household in Bihar was significantly lower than in Assam, possibly reflecting the significantly smaller herd size in Bihar. A study from Ethiopia reported better reproductive performance in smaller herds [27]. Our study found that larger herd size (>10 dairy animals) was significantly associated with higher rates of the selected reproductive problems (Table 1). Larger herds may have poorer sanitation and hygiene with large numbers of animals kept in small confined places where disease transmission might be easier [15]. Further, we found that households rearing animals under a fully stall-fed system (zero grazing) experienced higher rates of the selected reproductive problems than those who reared dairy animals under partly stall-fed conditions. Haile et al. (2014) reported a contrary finding with higher reproductive problems in partly stall-fed than in fully stall-fed conditions [4].

Further, the selected reproductive problems were relatively higher in urban than rural areas (Table 1) associated with higher concentrations of large sized farms in urban areas because of access to raw milk urban markets, particularly in Assam, where about 97% of milk is marketed through the informal dairy value chain [28]. Some recent studies reported higher seroprevalence of *Brucella* spp. and *Leptospira* spp. infection in urban/peri-urban areas than rural areas in Bihar and Assam [29,30,31]; these infections are considered as the main cause of the five selected reproductive problems [15,32,33,34]. 

Different researchers have reported different rates of prevalence of reproductive problems in different parts of the world, but most agree that the prevalence of the reproductive problems we selected ranges widely from 0.2% to 25.0%. These differences might be because of location of the farms (urban or rural areas), rearing systems, herd size (small, medium or large), and breed of animals (indigenous or improved) as discussed above [15]. Regional differences in etiology also cannot be ruled out [15,34]. We found that repeat breeding (23.2%), retained placenta (6.1%) and abortion (4.9%) were the most important of the five problems we studied. Incidence of purulent vaginal discharge (2.9%) and stillbirth (1.0%) cases was relatively lower in our study areas. Our findings are fully or partly in agreement with studies conducted in Meghalaya (India), Bangladesh, Kashmir (India), Haryana (India) and Ethiopia [4,6,7,22,23,35]. 

Among the costs caused by reproductive problems, we found that the highest cost was due to management of dairy animals during an extended calving interval. We found that repeat breeding and abortion resulted in loss of reproductive months in both Assam and Bihar. The mean inter-calving period reported in our study (15.2 months) was similar to that found in a study conducted in Bangladesh (15.3 months) [36] but slightly better than found in Pakistan (16.8 months), Ethiopia (17.9 months) and Tanzania (16.7 months) [25,37,38] while much worse than in countries like Korea (13.8 months) [39] or USA (13 months) [40]. More reproductive month loss from abortion was found in Assam than in Bihar, attributable to a larger proportion of abortions in the 3rd trimester. The different timing of abortion may be due to differing etiologies. Kamrup (Metropolitan) district of Assam, for example, has been reported to have a high prevalence of brucellosis [15,29,30], and studies suggest that dairy animals that suffer from brucellosis generally abort in third trimester [10,41,42]. 

Because of reproductive problems, dairy animals become less productive and more expensive to rear. Therefore, farmers report being compelled to sell the animals at lower value before the end of their productive life. This contributed to significant economic loss to the farmers. A study in Michigan found 60.8% of the repeat breeders were culled [43]. Consistent with these results, half of the repeat breeders in our study were found to be salvage-sold. A relatively lower salvage selling percentage (30.4%) for repeat breeders was reported by another study from India [44]. In India, culling of diseased animals is not easy as slaughtering of cows is prohibited by Act of law [45]. A strong sense of belongingness, socio-religious beliefs and policy environment also greatly influence dairy farmers in reluctance to cull diseased animals, and, perhaps, reluctance to report if they do sell. Poor knowledge of farmers about the infectious diseases like brucellosis, leptospirosis etc., and their preventive measures [14] may reduce the adoption of proper reproductive health management practices (e.g., proper feed, clean and hygiene, deworming, vaccination, timely breeding, timely treatment, culling, quarantine, salvage selling, etc.) in the studied states. Further, poor farm record keeping system followed by the farming community may limit their capacity to identify and judge the reproductive problems correctly and to take appropriate corrective measures on time. 

### 4.2. Economic Cost of Reproductive Problems

The terms ‘loss’ and ‘cost’ are often used rather loosely, and even interchangeably by many researchers [16] but in this paper the terms are used consistently as per the definition stated in the methodology section. A study in US estimated the economic cost of clinical diseases of dairy animals using stochastic simulation model based on seven parameters which include veterinarians’ fee, expenditure for medicine, expenditure for labour, loss of milk, loss of culling, loss of management of dairy animals during extended calving interval and on farm death [46]. In our study also we considered all the above expenditure and loss, although we did not find any case of morality of dairy animals because of reproductive problems. Several studies have highlighted significant economic costs in terms of reduced milk production, expenditure on medication, reduced calf production, prolonged calving interval and early depreciation of potentially useful cows with reproductive problems [10,18,47]. Our present study confirms the five selected reproductive problems lead to such economic costs and that costs are substantial. Among them, the highest cost was due to management of animals during an extended calving interval period followed by low salvage returns for culled animals. This ranking corroborates the findings of Dijukizen et al. [48], but differs from the findings of Patel et al. [17] who reported that reduced milk yield caused the highest cost. We did not find such a high economic cost because of reduced milk yield. This may be explained by milk yield loss being observed mainly in dairy animals that aborted in first trimester and early second trimester, whereas cost of managing an extended calving interval applied to every aborted animal and every repeat breeder. A study from Tanzania also reported longer inter-calving period as an important driver of economic loss [37]. 

The treatment expenditure reported by various researchers varies even within the country possibly because of variation in location of farms, time period, access to services, quality of services, type of treatment and type of individual engaged in treatment (including community animal health workers, veterinary diploma holders, veterinary graduates, veterinary professors and specialists): all have a potentially important bearing on the expenditure. Relatively lower expenditures for the treatment of abortion (INR 250/USD 3.7), repeat breeding (INR 506/USD 7.5) and retained placenta (INR 320/USD 4.8) were reported from Gujarat, India (2003–2005) [18] compared to our reported expenditure of INR 1319 (USD 19.6), INR 1638 (USD 24.4) and INR 761 (USD 11.3), respectively, for the same conditions. Similarly, lower treatment expenditure for reproductive problems (INR 750/USD 11.2) was reported by another researcher [17] in Gujarat, India, but this dates from an earlier time period and is not adjusted for inflation. 

Further, the expenditure estimated for treatment of reproductive problems in this study does not reflect the expenditure on treatment of all animals with the selected reproductive problems. We found that about one fifth of the dairy animals with reproductive problems were left untreated, which may be attributed to poor access to veterinary services, poor availability of veterinarians at night, lack of funds to treat animals, lack of skill of farmers to detect some problems or lack of knowledge about the effects of the problem [6]. A study from 2007 suggested that only about 32% of the farmers in India get access to services, and that services are mainly for curative rather than prophylactic treatment [49]. Moreover, of those farmers who availed veterinary services, 36.7% were not satisfied with the veterinary and extension services [50]. The need for good veterinary service with strong infrastructure, quality bulls or semen and need-based training and extension services have been identified as important elements for addressing reproductive health problems in Haryana, India [6]. 

Cost estimation of reproductive problems at state level is important to inform policymakers and to help them make evidence-based decisions for designing control programme. This study estimated an economic cost of INR 3963.1 (USD 59.0) million in Assam and INR 30,500.0 (USD 453.9) million in Bihar due to five selected reproductive problems. An economic estimation from India reported a loss of USD 3.4 billion to the livestock sector for the single disease brucellosis, of which 95.6% (USD 3.2 billion) was incurred in the dairy sector alone [51]. That study estimated that brucellosis caused a loss of USD 6.8 per cattle and USD 18.2 per buffalo which was less than our estimated cost of five reproductive problems (INR 2,424.9/USD 36.1) per animal. Another recent study in India estimated a total loss of INR 92,120 (USD 1370.8) million for the country because of brucellosis [52] which is again seems lower than our estimate. It seems quite obvious as they estimated the cost of reproductive problems only caused by brucellosis, however, we estimated the cost of reproductive problems irrespective of etiology. 

It may be worth mentioning here that we found a high incidence of reproductive problems in both Assam (32.9% animals) and Bihar (43.1%) but our previous seroprevalence studies on *Brucella* spp., *Leptospira* spp. and *Coxiella burnetii* in both the states (based on the same sampling frame as used for this study and on the same biological samples), found that seroprevalence of all three infectious agents greatly varied between the states. *Brucella* spp. seroprevalence at herd level in Assam was 16.5% in contrast to 0.3% in Bihar [15], while herd level *C. burnetii* seroprevalence in Assam was 5.8% in contrast to 27.1% in Bihar and *Leptospira* spp. seroprevalence in Assam was 1.2% in comparison to 4.5% in Bihar [31,34]. All three infectious agents may cause reproductive problems with a similar presentation in dairy animals [43,53,54] and therefore it may not be wise to estimate the economic cost based on investigation of one infectious agent (say brucellosis). Further, any economic estimation of reproductive problems based on a seroprevalence study without confirmatory diagnosis (based on historical, clinical, and laboratory investigation of the aetiology) could be highly misleading as seropositivity does not mean the disease is present. Therefore, in this manuscript we preferred not to assess the economic cost of reproductive problems based on specific pathogens but rather on syndromes.

The economic costs of reproductive problems can be expected to increase as dairy systems intensify and the costs of inputs and animals increase. Two estimates of annual cost per dairy animal for reproductive problems in the Netherlands of USD 80 [48] and USD 267 [55] are indeed much higher than the estimate reported in the present study (INR 2424.9/USD 36.1). Of course, they did not estimate the treatment expenditure of the five specific reproductive problems or culling/ salvage selling loss but estimated the economic cost of reduced milk yield, AI cost, calving management cost and increased calving interval cost. A more meaningful comparison is considering those costs relative to the gross production value of the animal, which equated to be 2% in the case of the second estimate from the Netherlands while the estimated costs of reproductive problems in our study are estimated to represent 4.1% of the mean value of dairy animals. 

The per animal cost of reproductive problems among the sampled households (Table 3) was found to be lower in Bihar (INR 4353/USD 65) than in Assam (INR 5207/USD 77) mainly because of lower cost of production, expenditure for treatment and price of milk in Bihar than in Assam. This might be because of the presence of a strong dairy cooperative system in Bihar (total cooperative members, n = 1,003,557 in 2015–2016) in comparison with Assam (total cooperative members, n = 15,817) [5] that supports the farmers in getting access to farm inputs at reasonable prices and selling the milk in bulk quantity relatively at lower price. In Assam, higher expenditure for management and treatment of cattle by farmers was reported mainly due to dependence of farmers on external supply of farm inputs (e.g., feed, AI semen, medicine etc.) mainly from the other parts of India which is more than 1000 km from away from Assam but closer to Bihar. Further, dairy animals are also more expensive in Assam than in Bihar because of the scarcity of improved animals owing to which, some dairy farmers in Assam import dairy animals from Bihar where the cost of dairy animals is relatively lower. 

In case of the extrapolated cost for the states, per animal cost was found about three times higher in Bihar (INR 2912.2/USD 43.3) compared to Assam (INR 1060.0/USD 15.8). This was mainly because of much higher improved dairy animal population (87.5%) in Bihar than in Assam among which reproductive problems were found significantly higher than in indigenous dairy animals. Again, the much higher (73.7%) dairy animal population in Bihar contributed to make the overall cost of reproductive problems higher (88.5%) in Bihar than in Assam. 

Under this study, we included the five most common reproductive problems which were identified by farmers using a syndromic approach. There are several other reproductive problems like anestrus, dystocia, metritis, endometritis, uterine prolapse etc. [8,24,56] but these are not included in our study. The key reason for focusing on selected reproductive problems rather than the full list of problems was that-some of the reproductive problems (repeat breeding, retained placenta, metritis, endo-metritis, purulent vaginal discharge, anoestrus etc.) were interrelated [9] and the underlying cause may be manifested in any of these, so their individual economic cost would be difficult to assess without proper laboratory investigation and farm records. As a result, the economic cost estimates presented here should be considered a lower bound, though the other diseases not included in our analysis are not expected to dramatically increase the overall estimated cost. Further, it could be mentioned that there is lack of uniformity in the reproductive problems included and items of cost estimation among different studies conducted by various researchers, therefore comparing the results of different studies is not straight forward. Again, in our study, we found some absent, or non-applicable, data mainly related to the cost of management of dairy animals. This was because farmers keep few management or day-to-day expenses record and often find it difficult to calculate out the cost as part of these expenses come from household feed resources or family labour.

## 5. Conclusions

It can be concluded that reproductive problems are common in both Assam and Bihar and are responsible annually for multi-million dollar costs to the dairy industry. By strengthening reproductive health management, the states can reduce this cost, estimated to account for approximately 4.1% value loss of dairy animals each year. This study has shown that the overall cost of reproductive problems contributed by a large indigenous dairy animal population is already high, and it could be higher as part of the indigenous population shifts to improve through cross-breeding programs. Therefore, more comprehensive efforts are required to reduce these costs. Further, in any future cost estimation study, it is important to make sure that both indigenous and improved animals are sufficiently represented to generate robust parameter estimates. This study opines that reproductive problems may manifest as a result of various infectious and non-infectious causes, and these could largely vary from place to place. Any economic estimation of reproductive problems based on assessment of any single causative agent (say brucellosis) may be highly misleading. In addition, economic estimation of reproductive problems based on any seroprevalence study, without confirmatory diagnosis of the aetiology, could lead to overestimation of the economic burden of the diseases as seropositivity does not mean occurrence of the disease. The study urges the need to investigate the causes behind the reproductive problems, and to explore the possible corrective measures that are appropriate according to the cause. Since one fifth of reproductive cases remain untreated, and culling or other disease preventive practices are poorly followed, the study suggests that there is a need to increase awareness and capacity among the farming communities to adopt better reproductive health management practices and to keep proper farm records that will help them to address the problems in a more timely and efficient manner. Our economic estimates may help policy makers to make appropriate investment decisions to increase access and quality of veterinary and extension services to dairy farmers. The significant differences among various attributes between Assam and Bihar indicate that the prevalence of reproductive problems and cost incurred varies from state to state and therefore a blanket approach for controlling reproductive problems may not work. More studies are required to investigate the economic cost of the full range of reproductive problems and to examine additional representative samples to extrapolate the cost for other states and the country as a whole.

## Figures and Tables

**Table 1:** Dairy farms with reproductive problems under different farm characteristics with level of significance between categories.

Farm Characteristics	Categories	No. of Dairy Farms that Reported the Occurrence of One or More Reproductive Problems/Corresponding Total (%)				
		Repeat Breeding	Abortion	Retained Placenta	Purulent Vaginal Discharge	Stillbirth
Location of the farms in states	Assam	59/242(24.4) **	35/242(14.5)	34/242(14.0)	26/242(10.7)	3/242 (1.2) *
	Bihar	132/292(45.4)	38/292(13.0)	39/292(13.3)	23/292(7.9)	12/292 (4.1)
Location of the farms in urban and rural areas	Rural	71/247(28.7) *	23/247(9.3) **	21/247(8.5) **	16/247(6.5) *	3/246 (1.2) *
	Urban	120/287(41.8)	50/285(17.5)	52/286(18.2)	33/286(11.5)	12/282 (4.3)
Farm size based on herd strength	Small (1–3 dairy animals)	99/306(32.3) **	18/305(5.9) **	25/305(8.2) **	10/305(3.3) **	5/304 (1.6) *
	Medium (4–10 dairy animals)	56/178(31.5)	27/177(15.2)	23/178(12.9)	19/178(10.7)	6/176 (3.4)
	Large (>10 dairy animals)	36/50(72.0)	28/50(56.0)	25/50(50.0)	20/50(40.0)	4/48 (8.3)
Rearing system followed	Fully stall-fed	165/316(52.2) **	64/315(20.3) **	65/315(20.6) **	43/315(13.6) **	14/310 (4.5) **
	Partly stall-fed	26/218(8.2)	9/217(4.1)	8/218(3.7)	6/218(2.7)	1/218 (0.5)
Breed of the animals kept	Indigenous	12/154(7.8) **	3/154(1.9) **	2/154(1.3) **	2/154(1.3) **	0/154 (0)
	Improved	179/380(47.1)	70/380(18.4)	71/380(18.7)	47/380(12.4)	15/380 (3.9)

* Significant (*p* ≤ 0.05) ** Highly significant (*p* ≤ 0.001).

**Table 2:** Parameters used for estimating the cost of selected reproductive problems ^a^ with reported positive observations/total observations (percentage) or mean value of the positive observations in the surveyed dairy farms of Assam and Bihar, *p*-value of inter-state variation and absent data (non-responding farms).

Parameters Used for Estimating the Cost of Reproductive Problems	Assam	Bihar	Total	*p*-Value	Non-Responding Farms
Mean number of dairy animals per farm	5.6 ± 0.6	3.3 ± 0.2	4.3 ± 0.3	<0.001	0
Farms with reproductive problems/total farms	68/242 (28.1) ^b^	151/292 (51.7)	219/534 (40.0)	<0.001	0
Dairy animals with reproductive problems/total dairy animals	444/1348 (32.9)	411/954 (43.1)	855/2302 (38.0)	<0.001	0
Mean reproductive problems in affected households	6.5 ± 0.7	2.7 ± 0.2	3.9 ± 0.3	<0.001	0
Abortion cases					
Farms with history of abortion in dairy animals/total farms	35/242 (14.5)	38/292 (13.0)	73/534 (13.8)	0.63	0
Dairy animals aborted in 1st trimester/total aborted dairy animals	4/62 (6.5)	4/50 (8.0)	8/112 (7.3)	0.04	0
Dairy animals aborted in 2nd trimester/total aborted dairy animals	9/62 (14.5)	24/50 (48.0)	33/112 (31.3)		0
Dairy animals aborted in 3rd trimester/total aborted dairy animals	49/62 (79.0)	22/50(44.0)	71/112 (61.5)		0
Aborted dairy animals/total dairy animals	62/1348 (4.6)	50/954 (5.2)	112/2302 (4.9)	0.05	0
Aborted animals treated/total aborted dairy animals	49/62 (79.0)	41/50 (82.0)	90/112 (80.5)	0.03	0
Mean treatment ^d^ expenditure ^c^ of treated abortions	1475.7 ± 100.4	1182.1 ± 79.6	1319.9 ± 65.4	0.02	0
Repeat breeding cases					
Farms with repeat breeding/total farms	59/242 (24.4)	132/292 (45.2)	191/534 (34.9)	<0.001	0
Dairy animals with repeat breeding/total dairy animals	241/1348 (17.9)	272/954 (28.5)	513/2302 (23.2)	<0.001	0
Dairy animals with repeat breeding treated/total dairy animals with repeat breeding	206/241 (85.5)	197/272 (72.4)	403/513 (79.0)	<0.001	0
Mean extra insemination required per repeat breeder	2.8 ± 0.1	2.5 ± 0.1	2.6 ± 0.1	0.09	0
Mean expenditure of inseminating or natural mating per time	200.5 ± 6.8	128.0 ± 2.8	150.0 ± 0.4	<0.001	120
Mean treatment ^e^ expenditure of treated repeat breeders (through 7 FGDs ^f^)	1750.1 ± 61.9	1542.8 ± 52.8	1638.5 ± 48.7	0.02	0
Retained placenta cases					
Farms with retained placenta/total farms	34/242 (14.0)	39/292 (13.3)	73/534 (13.7)	0.05	0
Dairy animals with retained placenta/total dairy animals	95/1348 (7.0)	49/954 (5.1)	144/2302 (6.1)	0.005	0
Mean dairy animals with retained placenta per farm reporting the problem	2.7 ± 0.5	1.2 ± 0.2	1.9 ± 0.3	0.005	0
Dairy animals with retained placenta treated/total dairy animals with retained placenta	79/95 (83.1)	37/49 (75.5)	116/144 (79.3)	0.03	0
Mean treatment ^g^ expenditure of treated retained placenta cases (through 7 FGDs)	825.0 ± 44.2	707.1 ± 35.2	761.5 ± 31.6	0.06	0
Purulent vaginal discharge cases					
Farms with purulent vaginal discharge cases/total farms	26/242 (10.7)	23/292 (7.9)	49/534 (9.3)	0.26	0
Dairy animals with purulent vaginal discharge/total dairy animals	41/1348 (3.0)	26/954(2.7)	67/2302 (2.9)	0.01	0
Dairy animals with purulent vaginal discharge treated/total dairy animals with purulent vaginal discharge	31/41 (75.6)	22/26 (84.6)	53/67 (80.1)	0.03	0
Mean dairy animals with purulent vaginal discharge per farm reporting the problem	1.6 ± 0.1	1.1 ± 0.1	1.4 ± 0.1	0.01	0
Mean treatment ^h^ expenditure of treated purulent vaginal discharge cases (through 7 FGDs)	1216.7 ± 70.3	1064.3 ± 44.6	1134.6 ± 44.3	0.08	0
Stillbirth cases					
Farms with stillbirth cases/total farms	3/242(1.2)	12/292 (4.1)	15/534 (2.7)	4.01	0
Dairy animals with stillbirth/total dairy animals	5/1348 (0.4)	14/954(1.5)	19/2302 (1.0)	0.09	0
Dairy animals with stillbirth treated/total dairy animals with stillbirth	4/5 (80.0)	7/14 (50.0)	11/19 (65.0)	0.62	0
Mean stillbirth cases per affected farm	1.7 ± 0.3	1.2 ± 0.1	1.3 ± 0.1	0.62	0
Mean treatment expenditure of treated stillbirth cases (through 7 FGDs)	1066.7 ± 49.4	957.1 ± 57.1	1007.7 ± 40.0	0.18	0
Reproductive month loss					
Mean calving interval (months)	15.5 ± 0.1	14.9 ± 0.1	15.2 ± 0.1	<0.001	482
Mean reproductive month loss because of abortion (months)	5.7 ± 0.5	4.9 ± 0.3	5.3 ± 0.3	0.02	0
Mean reproductive month loss because of repeat breeding (months)	1.9 ± 0.1	1.7 ± 0.6	1.8 ± 0.1	0.09	0
Expenditure of managing dairy animals					
Mean expenditure of concentrate feed consumption/dairy animal/day	70.8 ± 6.1	59.3 ± 2.8	64.6 ± 3.4	0.09	395
Mean expenditure of fodder consumption/dairy animal/day	20.0 ± 2.9	15.4 ± 1.0	17.5 ± 1.5	0.14	406
Mean expenditure of other miscellaneous items (medicine, breeding, detergents, etc.)/dairy animal/day	12.0 ± 0.5	10.6 ± 0.4	11.2 ± 0.5	0.18	413
Mean labour expenditure/animal/day	13.7 ± 1.3	12.3 ± 0.5	12.9 ± 0.7	0.30	413
Mean electricity expenditure/animal/day	1.7 ± 0.4	1.9 ± 0.2	1.8 ± 0.2	0.68	413
Total expenditure of management/animal/day	118.2 ± 11.5	99.4 ± 4.0	108.1 ± 6.1	0.12	413
Reduced milk yield of aborted animals					
No. of animals suffered milk yield loss	34	37	35.5	0.52	0
Farms with reduced milk yield	20	27	23.5	0.41	0
Mean volume of milk loss per animal/day (litre)	1.8 ± 0.2	1.3 ± 0.1	1.6 ± 0.1	0.07	0
Mean days with reduced milk yield loss/aborted animal	104.0 ± 5.5	73.3 ± 2.3	88.6 ± 4.50	0.04	0
Mean milk price/litre at the farm gate	41.3 ± 0.4	29.8 ± 0.4	33.8 ± 0.4	0.001	145
Salvage selling					
Farms that sold dairy animals for salvage/total farms	32/242 (13.2)	26/292 (8.9)	58/534 (11.0)	0.11	0
Dairy animals that were sold for salvage/total dairy animals	57/1348 (4.2)	35/954 (3.7)	92/2302 (3.9)	0.10	0
Mean price of healthy animal	65,468.7 ± 4401.5	50,961.5 ± 4132.6	58,965.5 ± 3175.5	0.40	0
Mean price of animals with reproductive problems	14,281.2 ± 1335.9	12,846.1 ± 929.3	13,637.9 ± 845.1	0.02	0
Mean salvages selling loss per dairy animal	51,187.5 ± 3849.1	38,115.4 ± 3640.5	45,327.6 ± 2791.5	0.02	0

^a^ Here reproductive problems mean incidence of the five major reproduction related problems (abortion, repeat breeding, retained placenta, purulent vaginal discharge and stillbirth) of large ruminant dairy animals occurred in the previous 12 months from the date of survey. ^b^ Figure in the parenthesis indicates percentage of the corresponding total. ^c^ All financial figures are in Indian Rupees (INR). Conversion rate USD 1 = INR 67.2 (average conversion rate of the year 2016, the year of study. Source—https://www.exchangerates.org.uk/USD-INR-spot-exchange-rates-history-2016.html, accessed on 17 May 2021). ^d^ Treatment of abortion and stillbirth included antibiotic/hormone/vitamin/others depending on the complexity of the problem. ^e^ Treatment of repeat breeding included antibiotic/hormone/dewormer/mineral mixture/irrigation of uterus with antiseptic solution/others depending on the possible cause of repeat breeding. ^f^ In every community development block (CDB) one focus group discussion (FGD) was conducted with the farmers who encountered the reproductive problems to get the agreed cost of each problem, where all total 121 farmers participated. ^g^ Treatment of retained placenta included hormone/manual removal of placenta/irrigation of uterus with antiseptic solution/antibiotic/others depending on the complexity. ^h^ Treatment of purulent vaginal discharge included antibiotic/irrigation of uterus with antiseptic solution/others depending on the complexity.

**Table 3:** Estimated cost of reproductive problems among the surveyed dairy farming households in Assam and Bihar.

Item of Cost of Reproductive Problems	Total Estimated Cost in Indian Rupees (INR) of the Affected Animals in Surveyed Farming Households (All Figures Are in Thousands)						Cost per Animal (INR) ^c^		% of the Cost	
	Assam			Bihar			Assam		Bihar	
	Indi. ^a^	Impro. ^b^	Total	Indi.	Impro.	Total				
Treatment of aborted animals	2.9	69.4	72.3	1.2	47.3	48.5	54	51	1.0	1.2
Extra inseminations of repeat breeders	3.4	131.9	135.3	3.8	83.2	87.0	100	91	1.9	2.1
Treatment of repeat breeding cases	10.5	350.0	360.5	9.3	294.7	303.9	267	319	5.1	7.3
Treatment of retained placenta cases	2.5	62.7	65.2	0	26.2	26.2	48	27	0.9	0.6
Treatment of purulent vaginal discharge cases	1.2	36.5	37.7	0	23.4	23.4	28	25	0.5	0.6
Treatment of stillbirth cases	0	4.3	4.3	0	6.7	6.7	3	7	0.1	0.2
Management of animal for extra reproductive month loss because of abortion	40.4	1212.7	1253.1	14.6	716.7	731.3	930	767	17.9	17.6
Management of animal for extra reproductive month loss because of repeat breeding	42.5	1666.6	1709.2	64.5	1397.0	1461.4	1268	1532	24.4	35.2
Loss of milk production	15.5	448.4	463.9	2.8	127.8	130.6	344	137	6.6	3.1
Loss of salvage selling	51,187	2866.5	2917.7	38.1	1295.9	1334.0	2164	1398	41.6	32.1
Total cost among surveyed animals (in INR)	170,138	6849.0	7019.1	134.3	4018.8	4153.1	5207	4353	100	100
Total cost among surveyed animals (in USD) ^d^	2532	101.9	104.4	2.0	59.8	61.8	78	65		

^a^ Indigenous (native breed or non-descript), ^b^ Improved (exotic breed or cross-bred), ^c^ Per animal cost among the surveyed animals in Assam (*n* = 1348) and Bihar (*n* = 954) ^d^ Conversation rate USD 1 = INR 67.2 (average of the year in 2016, Source—https://www.exchangerates.org.uk/USD-INR-spot-exchange-rates-history-2016.html, accessed on 17 May 2021).

**Table 4:** Percent dairy animals affected in surveyed areas and estimated numbers of total dairy animals affected in the whole state.

Item of Cost of Reproductive Problems	Percent Dairy Animals Affected in the Surveyed Areas of the State				Estimated Total Number of Dairy Animals Affected in the Whole State (Assuming the Same Percentage of the Surveyed Areas for the States)					
	Assam		Bihar		Assam			Bihar		
	Indi. ^a^	Impro. ^b^	Indi.	Impro.	Indi.	Impro.	Total Assam	Indi.	Impro.	Total Bihar
Animal treated for abortion	0.7	4.4	1.9	4.4	24,436	12,931	37,367	158,867	91,155	250,022
Repeat breeders bred through extra AI	2.1	22.1	22.6	28.9	73,308	64,656	137,964	1,906,406	592,509	2,498,915
Animals treated for repeat breeding	2.1	18.8	11.3	21.2	73,308	55,027	128,335	953,203	435,266	1,388,469
Animals treated for retained placenta	1.1	7.1	-	4.1	36,654	20,910	57,564	-	84,319	84,319
Animals treated for Purulent vaginal discharge	0.3	2.8	-	2.4	12,218	8254	20,472	-	50,135	50,135
Animals treated for Stillbirth	-	0.4	-	0.8	-	1101	1101	-	15,952	15,952
Animals managed for extra reproductive months caused by abortion	0.7	5.6	1.9	5.4	24,436	16,508	40,944	158,867	111,665	270,532
Animals managed for extra reproductive months caused by repeat breeding	2.1	22.1	22.6	28.9	73,308	64,656	137,964	1,906,406	592,509	2,498,915
Animals faced reduced milk yield	0.7	5.4	1.9	5.0	24,436	15,958	40,394	158,867	102,550	261,417
Animals sold under lower salvage value	0.3	5.3	1.9	3.8	12,218	15,407	27,625	158,867	77,482	236,349

^a^ Indigenous (native breed/non-descript), ^b^ Improved (exotic or cross breed).

**Table 5:** Estimated cost of selected reproductive problems in the states of Assam and Bihar (all figures are in Million).

Item of Cost of Reproductive Problems	Estimated Cost in Assam in Indian Rupees (INR)			Estimated Cost in Bihar in Indian Rupees (INR)			Total of Assam and Bihar
	Indi. ^a^	Impro. ^b^	Total	Indi.	Impro.	Total	
Treatment of aborted animals	36.1	19.1	55.1	187.8	107.8	295.6	350.7
Extra inseminations of repeat breeders	41.2	36.3	77.4	61.0	189.6	799.6	877.1
Treatment of repeat breeding cases	128.3	96.3	224.6	1470.6	671.5	2142.1	236.7
Treatment of retained placenta cases	30.2	17.2	47.5	0	59.6	59.6	107.1
Treatment of purulent vaginal discharge cases	14.9	10.0	24.9	0	53.4	53.4	78/3
Treatment of stillbirth cases	0	1.2	1.2	0	15.3	15.3	16.4
Management of animal for extra reproductive month loss because of abortion	493.9	333.7	827.7	2323.6	1633.2	3956.8	4784.4
Management of animal for extra reproductive month loss because of repeat breeding	519.9	458.5	978.4	10,243.1	3183.5	13,426.7	14,405.1
Loss of milk production	188.9	123.4	312.3	451.2	291.2	742.4	1054.7
Loss of salvage selling	625.4	788.7	1414.1	6055.2	2953.2	9008.4	10,422.5
Total cost among surveyed animals (in INR)	2078.7	1884.4	3963.1	21,341.6	9158.4	30,500.0	34,463.1
Total cost (in USD) ^c^	30.9	28.0	59.0	317.6	136.3	453.9	512.8

^a^ Indigenous (native breed or non-descript indigenous), ^b^ Improved (exotic or cross breed) ^c^ Conversation rate USD 1 = INR 67.2 (average of the year in 2016, Source—https://www.exchangerates.org.uk/USD-INR-spot-exchange-rates-history-2016.html, accessed on 17 May 2021).

## Data Availability

Data will be made available by the authors upon request.
